# Cough reflex sensitivity and urge-to-cough deterioration in dementia with Lewy bodies

**DOI:** 10.1183/23120541.00108-2019

**Published:** 2020-03-09

**Authors:** Takae Ebihara, Peijun Gui, Chika Ooyama, Koichi Kozaki, Satoru Ebihara

**Affiliations:** 1 Dept of Geriatric Medicine, Kyorin University School of Medicine, Tokyo, Japan; 2 Dept of Internal Medicine and Rehabilitation Science, Tohoku University Graduate School of Medicine, Sendai, Japan; 3 Dept of Rehabilitation Medicine, Beijing Friendship Hospital, Capital Medical University, Beijing, China; 4 Dept of Rehabilitation Medicine, Toho University Graduate School of Medicine, Tokyo, Japan

## Abstract

Cough, an important respiratory symptom, predominantly involves the brainstem and the urge-to-cough (UTC) is modulated by the cerebral cortex. Lewy body disease is associated with decreased cough reflex sensitivity and central respiratory chemosensitivity. Additionally, the insula, associated with the UTC, shows decreased activation and atrophy in dementia with Lewy bodies (DLB). We investigated the relationships between cognition and cough reflex and the UTC and compared the differences in responses of patients with DLB and other dementia subtypes.

We conducted a cross-sectional study within a geriatric ward of a university hospital involving elderly patients diagnosed with Alzheimer's disease (AD), DLB, or non-dementia (controls). The cough reflex sensitivities were estimated based on the lowest concentrations of inhaled citric acid that could induce ≥2 coughs (C_2_) or ≥5 coughs (C_5_). Subjects were asked to rate the UTC based on the threshold concentrations (C_u_) using the modified Borg scale.

C_2_, C_5_ and C_u_ were negatively correlated with cognitive function in female participants but not in males (p<0.01). The cough reflex sensitivities expressed as C_2_ and C_5_ were significantly higher in the DLB group than in the AD and control groups (p<0.01 adjusted for gender). The UTC threshold expressed as C_u_ was also significantly higher in the DLB group, while the UTC log–log slope was less responsive in the DLB group than in the other groups.

The cough reflex sensitivity and perceived UTC deteriorated in the DLB group more than in the other groups. This result might be valuable in treating patients with DLB.

## Abstract 2

**Morbidity and mortality of aspiration pneumonia (AP) in people with dementia are increasing. The cough reflex sensitivity and the urge to cough, which are important responsible factors for AP, are deteriorated in dementia with Lewy bodies.** http://bit.ly/2Yyax9w

## Introduction

Cough is a crucial response function of the respiratory defence mechanism and involves the brainstem, where information from sensory nerve endings is processed in response to various stimuli. The urge-to-cough (UTC) is a sensation that plays an essential role in both initiating and inhibiting reflexive cough [1]. The UTC as a cognitive cough sensation, mediated by the cerebral cortex or the subcortical regions, occurs at a concentration threshold of cough-evoking stimuli lower than that required to evoke a motor cough [2]. Recently, the UTC was reported to activate multiple brain regions such as the insula, anterior midcingulate cortex, primary sensory cortex, orbitofrontal cortex, supplementary motor area and cerebellum [2, 3].

Therefore, the occurrence of cerebrovascular disease should impede the control of the modified pathway from the upper cough centre to the cough reflex arch, resulting in an elevated threshold for the cough reflex evoked by citric acid inhalation. Based on this hypothesis we previously reported decreased cough reflex sensitivity, as well as diminished sense of UTC, in patients with cerebrovascular diseases who suffered from recurrent aspiration pneumonia (AP) [4, 5]. Moreover, we suggested that this might explain the impairment of the central cough network ranging from the medullary to the cortices in patients with cerebrovascular disease and also their susceptibility to pneumonia.

The Japanese government predicts a sharp increase in the number of patients with dementia to 7 million in 2025 [6]. Both morbidity and mortality associated with pneumonia increases with age as well as in patients with advanced dementia [7]. A recent report showed that pneumonia complications were observed at high rates in dementia with Lewy bodies (DLB) [8]. Previous studies have observed reduced ventilatory response to hypercapnia in patients with DLB [9] and decreased chemosensitivity to hypoxia and hypercapnia (as well as decreased perception of dyspnoea and cough reflex sensitivity) in patients with Parkinson's disease (PD), which, along with DLB, form part of the Lewy body disease (LBD) spectrum [10, 11]. Furthermore, cerebral imaging research has revealed that the insular cortex, where the UTC is activated, shows bilateral hypoperfusion in patients with recurrent pneumonia and atrophy as well as hypoperfusion in patients with prodromal or clinical DLB [12–14].

As such, we hypothesised that the cough cortical sensation, which could influence the onset or exacerbation of pneumonia, is attenuated in dementia patients and particularly in those with DLB.

To our knowledge, no previous study has investigated cough reflex sensitivity and UTC in elderly patients with or without dementia as well as the type of dementia. Thus, we examined the relationships between cognitive function, cough reflex and UTC in patients with and without dementia.

## Methods

### Subjects and study protocol

**FIGURE 1:**
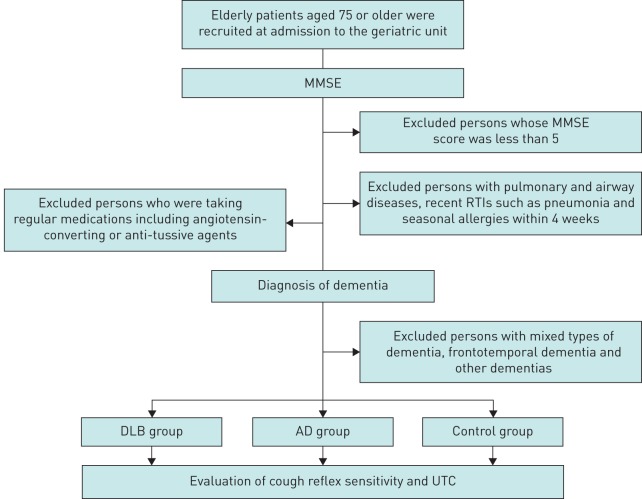
Flow diagram describing the study protocol. MMSE: mini mental state examination; RTI: respiratory tract infection; DLB: dementia with Lewy bodies; AD: Alzheimer's disease; UTC: urge-to-cough.

Study participants were recruited from among patients admitted to the geriatric unit of Tohoku University Hospital from April 2014 to March 2015 (figure 1). A cross-sectional study was conducted among all the elderly patients aged 75 years or older in the unit. Patients with a mini mental state examination (MMSE) score [15] of less than five were excluded, as were those with a form of dementia other than DLB and Alzheimer's disease (AD), including cerebrovascular-type dementia, mixed types of dementia and frontotemporal dementia amongst others. In addition, patients were without a history of pulmonary and airway diseases, recent suggestive symptoms (within 4 weeks), respiratory tract infections (RTIs) and seasonal allergies. Subjects did not take any regular medication including angiotensin-converting agents or anti-tussive agents. Finally, a total of 71 patients were included in the study, which consisted of 19 patients with DLB (the DLB group), 30 patients with AD (the AD group) and 22 patients without dementia (MMSE >25, the control group).

### Dementia diagnosis

Dementia was diagnosed based on the cognitive decline measured by a neuropsychological test battery and observation of impairments in social or occupational function. AD and DLB were diagnosed according to the NINCDS-ADRDA criteria [16] and the McKeith criteria [17, 18], respectively, in combination with cerebral imaging performed by at least one neurologist and one neuropsychologist. We used magnetic resonance imaging (MRI) to investigate structural changes in brain tissue and a software program using the voxel-based specific regional analysis system, the SPM package (Wellcome Centre for Human Neuroimaging, www.fil.ion.ucl.c.uk/spm/), was used in examining the degree of atrophy in the hippocampus in the diagnosis of patients with AD [19]. Single-photon emission computed tomography (CT) was also used as it detects changes in the bloodstream in specific regions of the brain, which is useful in the diagnosis of AD or DLB. Furthermore, as an auxiliary measure for the diagnosis of DLB, ^123^I-metaiodobenzylguanidine myocardial scintigraphy was used to detect autonomic nerve dysfunction by calculating the heart-to-mediastinum (H/M) uptake ratio in early and delayed phases of DLB (cut off=1.56) [17, 18]. The H/M uptake ratio in the DLB group was 1.54±0.28.

### Evaluation of cough reflex sensitivity and UTC

Before examining cough reflex and UTC, patients were assessed to have been in a stable condition for >3 months without developing infectious diseases, gastroesophageal reflux or allergic reactions and without receiving medications such as angiotensin-converting enzyme (ACE) inhibitors.

Simple standard instructions were given to each patient. Citric acid was used in the evaluation of cough reflex sensitivity as it had been used in our previous study for the observation of depressed cough in elderly patients [5]. Evaluation was conducted with a tidal breathing nebulised solution using an ultrasonic nebuliser (MU-32, Sharp Co. Ltd., Osaka, Japan), which could generated particles with a mean mass median diameter of 5.4 μm at an output of 2.2 mL·min^−1^. First, subjects inhaled the physiological saline solution using an ultrasonic nebuliser as a control phase. Subsequently, citric acid dissolved in saline was provided at two-fold incremental concentrations from 0.7 to 360 mg·mL^−1^. The number of coughs was counted by laboratory technicians who were blinded to the clinical details of patients and the purpose of the study. This was done both audibly as the “cough sound” and visually. Solutions with an incremental concentration of citric acid were inhaled until five or more coughs were elicited. A 2-min interval separated each nebuliser application. The cough reflex sensitivity was estimated by measuring the lowest concentration of citric acid that was able to provoke two or more coughs (C_2_) and five or more coughs (C_5_). Immediately after the completion of each nebuliser application, the subjects were asked to rate the level of their UTC. The modified Borg scale was used to allow subjects to rate UTC [5]. The scale ranged from zero (no UTC) to 10 (maximum UTC). The UTC scale was placed in front of the subjects and they pointed at the appropriate number, which was then recorded by the experimenter. The intensity of the UTC was assessed by log C_u_ (where Cu=concentration of citric acid at a threshold of UTC). To evaluate UTC, subjects were requested to ignore sensations other than the UTC itself, including those of dyspnoea, burning, irritation, choking and smoke in the throat. Subjects were warned that their sensation of the UTC would be either intensified, attenuated or would remain the same during the citric acid challenges and that their rating on the modified Borg scale should reflect this.

### Institutional review board

All clinical and instrumental examinations were performed in a university hospital setting as part of clinical care for patients with neurodegenerative disease. This study was conducted in accordance with the regulations of the Institutional Review Board and the Declaration of Helsinki. Written informed consent was obtained from all patients and their data were treated under the local privacy rules and regulations.

### Data analysis

Data are expressed as mean±sd unless otherwise stated. Age, body mass index (BMI), Brinkman index, pulmonary function and arterial blood gas levels were compared among the AD group, the DLB group and the control group *via* one-way ANOVA using SPSS version 22.0 (IBM Co., Japan). Moreover, one-way ANOVA was used to compare the concentrations of citric acid (C_2_, C_5_ and C_u_) and the UTC log–log scale among the three groups. Furthermore, these variables were analysed *via* two-way ANOVA to avoid the effect of gender differences in the groups compared. Spearman's correlation test was used to analyse the correlation between cognitive function, the concentration of citric acid (C_2_, C_5_ and C_u_) and the UTC on a log–log scale. A p-value of less than 0.05 was considered statistically significant.

The sensitivity and specificity of the citric acid challenge test for distinguishing patients with DLB from patients with AD or those without dementia (controls) were determined using receiver operating characteristic (ROC) curve analysis. Youden's index was used to identify the optimal threshold [20]. According to this index, the optimal citric acid cough reflex threshold (C_5_) to distinguish patients with DLB from those with AD or those without dementia was >16.85 g·L^−1^ (sensitivity=68.4% and specificity=69.2%). The optimal UTC threshold (C_u_) was >1.05 g·L^−1^ (sensitivity=100% and specificity=31.4%).

To determine the sample size for the study, a pilot trial of five subjects in each group was executed to evaluate both cough reflex sensitivity and UTC. G*Power 3.1.8 [21] was used to calculate the sample size. Based on the results of the pilot trial, to achieve 95% power to detect differences among the means *versus* the alternative of equal means using an F test with a 0.05 significance level, a minimum sample of 12 subjects per group was required. We finally recruited 19 patients with DLB, 30 patients with AD and 22 patients without dementia.

## Results

Table 1 shows significant gender differences in age, Brinkman index and forced expiratory volume in 1 s (FEV_1_)/forced vital capacity (FVC) ratio (p<0.01, as analysed by Mann–Whitney test), while table 2 shows no significant gender differences in C_2_, C_5_, C_u_ and UTC slope. Moreover, no significant differences were found in terms of age, BMI, Brinkman index, lung function or arterial blood gas levels between the control, AD and DLB groups. The MMSE score for the control group was significantly higher than for the AD and DLB groups (27.82±2.24 *versus* 19.69±4.50 and 19.25±6.25, respectively; p<0.001, as analysed by one-way ANOVA; p_1_<0.001, as analysed by two-way ANOVA to adjust for gender) (table 3).

**TABLE 1:** Characteristics of patients stratified by sex

**Characteristic**	**Male**	**Female**	**p*-*value**
**Participants n**	32	39	
**Dementia type n**			
Control	14	8	
AD	9	21	
DLB	9	10	
**Age years**	76.47±8.27	80.24±5.62	**0.02**
**BMI kg·m^−2^**	22.10±3.20	21.31±3.24	0.34
**Brinkman Index**	413.40±430.78	13.57±44.28	**<0.01**
**MMSE score**	23.19±6.19	20.81±5.45	0.06
**FEV_1_ % predicted**	105.71±27.30	114.57±30.95	0.19
**FVC % predicted**	91.36±18.93	91.66±24.46	0.68
**FEV_1_/FVC %**	75.56±10.80	80.21±13.24	**0.04**
**FEV_1_/FVC % predicted**	118.91±15.86	112.50±18.58	0.1
**pH**	7.41±0.03	7.41±0.02	0.92
***P*_aO__2_** **mmHg**	86.94±13.74	87.72±7.95	0.39
***P*_aCO__2_** **mmHg**	39.10±3.90	39.86±3.40	0.58
**HCO_3_^−^ mmol·L^−1^**	24.48±2.76	24.97±2.20	0.34

Data are presented as mean±sd unless otherwise stated and were analysed using the Mann–Whitney test. p-Values in bold express significant difference (p<0.05). AD: Alzheimer's disease; DLB: dementia with Lewy bodies; BMI: body mass index; MMSE: mini mental state examination; FEV_1_: forced expiratory volume in 1 s; FVC: forced vital capacity; *P*_aO_2__: arterial oxygen tension; *P*_aCO_2__: arterial carbon dioxide tension.

**TABLE 2:** Comparison of cough between male and female subjects

**Characteristic**	**Male**	**Female**	**p-value**
**Patients n**	32	39	
**Dementia type n**			
Control	14	8	
AD	9	21	
DLB	9	10	
**C_2_ log g·L^−1^**	0.97±0.47	0.90±0.52	0.27
**C_5_ log g·L^−1^**	1.16±0.51	1.10±0.49	0.5
**C_u_ log g·L^−1^**	0.40±0.43	0.42±0.49	0.9
**UTC slope**	1.00±0.66	1.03±0.72	0.99

Data are presented as mean±sd unless otherwise stated and were analysed using the Mann–Whitney test. p-Values less than 0.05 were deemed to represent significant difference. AD: Alzheimer's disease; DLB: dementia with Lewy bodies; C_x_: lowest concentration of inhaled citric acid that could induce ≥x coughs; C_u_: concentration of citric acid at a threshold of UTC; UTC: urge-to-cough.

**TABLE 3:** Patients characteristics in the three study groups

**Characteristic**	**Control**	**AD**	**DLB**	**p-value**	**p_1_-value**
**Patients n**	22	30	19		
**Sex (M/F) n/n**	14/8	9/21	9/10		
**Age years**	76.64±6.37	80.40±6.43	77.67±8.70	0.146	0.257
**BMI kg·m^−2^**	22.08±3.53	21.62±2.92	21.26±3.45	0.732	0.852
**Brinkman Index**	340.12±462.08	110.19±221.91	146.63±323.86	0.063	0.349
**MMSE score**	27.82±2.24	19.69±4.50	19.25±6.25	**<0.001**	**<0.001**
**FEV_1_ % predicted**	109.66±19.91	119.33±33.03	95.48±29.62	**0.039**	0.117
**FVC % predicted**	91.65±22.25	98.18±19.93	79.33±20.66	**0.025**	0.064
**FEV_1_/FVC %**	80.91±10.66	75.86±12.10	77.72±14.65	0.363	0.095
**FEV_1_/FVC % predicted**	121.37±12.89	110.70±17.15	115.56±22.07	0.104	0.151
**pH**	7.41±0.02	7.41±0.02	7.42±0.03	0.193	0.076
***P*_aO__2_** **mmHg**	89.06±10.12	85.81±11.59	88.30±10.46	0.567	0.514
***P*_aCO__2_** **mmHg**	39.57±4.18	39.29±3.90	39.89±2.02	0.879	0.683
**HCO_3_^−^ mmol·L^−1^**	24.44±1.62	24.56±2.87	25.58±2.39	0.389	0.353

Data are presented as mean±sd unless otherwise stated and were analysed using one-way ANOVA (p-values) or using two-way ANOVA when adjusting for gender (p_1_-values). Values are in bold show where significant differences were expressed (p<0.05 or p_1_<0.05). AD: Alzheimer's disease; DLB: dementia with Lewy bodies; BMI: body mass index; MMSE: mini mental state examination; FEV_1_: forced expiratory volume in 1 s; FVC: forced vital capacity; *P*_aO_2__: arterial oxygen tension; *P*_aCO_2__: arterial carbon dioxide tension.

### Relationship between cognitive function and cough reflex sensitivity

**FIGURE 2:**
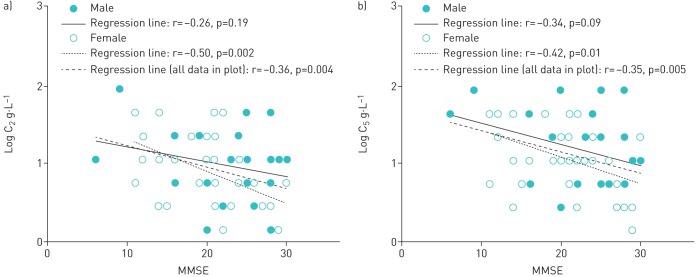
Relationship between cognitive function and the cough reflex sensitivity, showing significant differences between a) the relationship between mini mental state examination (MMSE) score and log C_2_ (where C_2_=lowest concentration of inhaled citric acid that could induce ≥2 coughs) and b) the relationship between MMSE and log C_5_ (where C_5_=lowest concentration of inhaled citric acid that could induce ≥5 coughs).

There was a significant correlation between the logarithm of citric acid concentration that was able to evoke two or more coughs (log C_2_) and MMSE (r=−0.36, p=0.004; as analysed by Pearson's correlation) (figure 2a). In particular, there was a significant correlation between C_2_ and MMSE in female participants but not in males (r=−0.50, p=0.002 *versus* r=−0.26, p=0.19; as analysed by Pearson's correlation).

Figure 2b shows a significant correlation between the logarithm of citric acid concentration that was able to evoke five or more coughs (log C_5_) and MMSE (r=−0.35, p=0.005; as analysed by Pearson's correlation). In a similar fashion to C_2_, a significant difference was found in the relationship between cognitive function and C_5_ in females but not in males (r=−0.42, p=0.01 *versus* r=−0.34, p=0.08; as analysed by Pearson's correlation).

### Comparison of cough reflex sensitivity among the three groups

**FIGURE 3:**
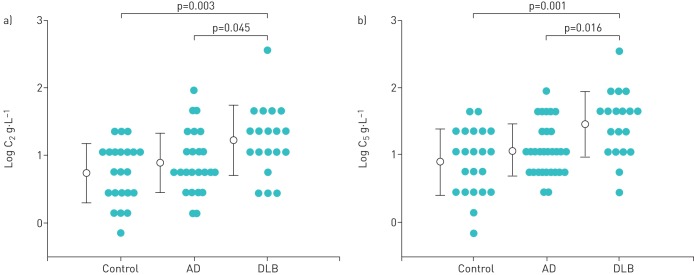
Comparison of cough reflex sensitivity among the control group, the Alzheimer's disease (AD) group and the dementia with Lewy bodies (DLB) group, showing: (a) the relationship of log C_2_ (where C_2_=lowest concentration of inhaled citric acid that could induce ≥2 coughs) for each group; (b) the relationship of log C_5_ (where C_5_=lowest concentration of inhaled citric acid that could induce ≥5 coughs) with each group. Two-way ANOVA showed significant differences in log C_2_ and log C_5_ between the DLB group and the control group or the AD group (p_1_=0.002 (a) and p_1_=0.0004 (b), as adjusted for gender). *Post hoc* tests showed significant differences in log C_2_ and log C_5_ between the DLB group and the control group or the AD group (p=0.003 and p=0.045, respectively (a) and p=0.001 and p=0.016, respectively (b), as adjusted for gender)

C_2_ was 0.73±0.44, 0.89±0.44 and 1.22±0.52 log g·L^−1^ for the control, AD and DLB groups, respectively. C_2_ was significantly higher for the DLB group than for the control group (p=0.003, as analysed by one-way ANOVA; p_1_=0.002, as analysed by two-way ANOVA to adjust for gender) and the AD group (p=0.045) (table 4 and figure 3a); however, there was no significant difference in C_2_ between the AD group and the control group (p=0.7). C_5_ was 0.89±0.49, 1.07±0.39 and 1.46±0.49 log g·L^−1^for the control, AD and DLB groups, respectively. C_5_ was significantly higher in the DLB group than in the control group (p=0.001, as analysed by one-way ANOVA; p_1_=0.0004, as analysed by two-way ANOVA to adjust for gender) and the AD group (p=0.016); however, there was no significant difference between the AD and control groups (p=0.48) (table 4 and figure 3b).

**TABLE 4:** Comparison of cough among the three study groups

**Characteristic**	**Control**	**AD**	**DLB**	**p-value**	**p_1_-value**
**Patients n**	22	30	19		
**Sex (M/F) n/n**	14/8	9/21	9/10		
**C_2_ log g·L^−1^**	0.73±0.44	0.89±0.44	1.22±0.52	**0.003**	**0.002**
**C_5_ log g·L^−1^**	0.89±0.49	1.07±0.39	1.46±0.49	**0.001**	**0.0004**
**C_u_ log g·L^−1^**	0.24±0.45	0.36±0.37	0.67±0.52	**0.008**	**0.004**
**UTC slope**	1.23±0.70	1.02±0.78	0.75±0.40	0.083	0.116

Data are presented as mean±sd and were analysed using one-way ANOVA (p-values) or using two-way ANOVA when adjusting for gender (p_1_-values). Values in bold show where significant differences were expressed (p<0.05 or p_1_<0.05). AD: Alzheimer's disease; DLB: dementia with Lewy bodies; C_x_: lowest concentration of inhaled citric acid that could induce ≥x coughs; C_u_: concentration of citric acid at a threshold of UTC; UTC: urge-to-cough.

### Relationship between cognitive function and UTC

**FIGURE 4:**
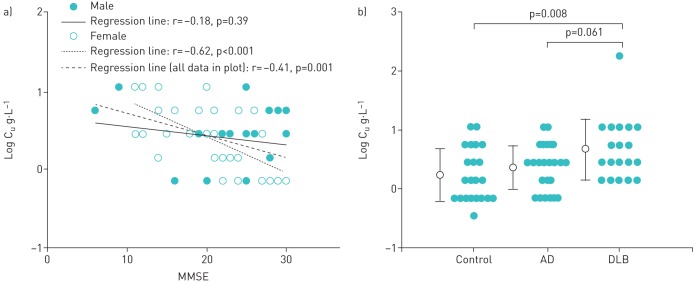
Relationship between cognitive function and the urge-to-cough (UTC), showing: a) the relationship between mini mental state examination (MMSE) score and log C_u_ (where C_u_=concentration of citric acid at a threshold of UTC), which illustrates significant differences; b) a comparison of log C_u_ between the control group, the Alzheimer's disease (AD) group and the dementia with Lewy bodies (DLB) group. Log C_u_ in the DLB group was significantly higher than in the control group and the AD group (p_1_=0.004, as adjusted for gender). *Post hoc* tests showed significant differences in log C_u_ between the DLB group and the control group or the AD group (p=0.008 and p=0.061, respectively).

Log C_u_ showed significant correlation with MMSE (r=−0.41, p=0.001) (figure 4a). In a similar fashion to C_2_ and C_5_, a significant difference in the relationship between cognitive function and C_u_ was observed in females but not in males (r=−0.62, p<0.001 *versus* r=−0.18, p=0.39; as analysed by Pearson's correlation).

### Comparison of UTC among the three groups

C_u_ was 0.67±0.52, 0.36±0.37 and 0.24±0.45 log g·L^−1^ for the DLB, AD and control groups, respectively. C_u_ was significantly higher for the DLB group than for the control group and the AD group (p=0.008, as analysed by one-way ANOVA; p_1_=0.004, as analysed by two-way ANOVA to adjust for gender) (table 4 and figure 4b); however, *post hoc* tests showed no significant difference for C_u_ between the DLB and AD groups (p=0.061).

### Relationship between cognitive function and UTC log–log slope

**FIGURE 5:**
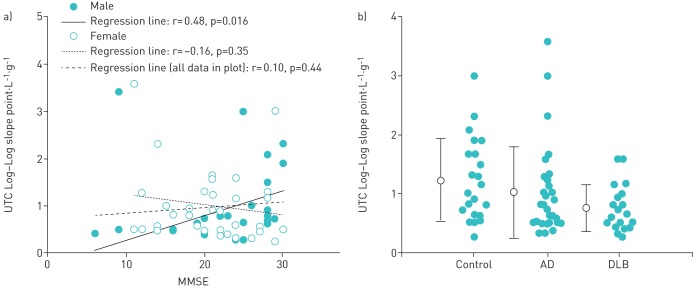
Relationship between (a) mini mental state examination (MMSE) score and the urge-to-cough (UTC) log–log scale slope of log C_u_ (where C_u_=concentration of citric acid at a threshold of UTC), which illustrates significant differences; (b) comparison of the UTC log–log scale slope of log C_u_ among the control group, the Alzheimer's disease (AD) group and the dementia with Lewy bodies (DLB) group. There was no significant difference amongst the three groups (p=0.111).

For each subject, the rated UTC scores were plotted against the corresponding citric acid concentrations using a log–log transformation. Since it is known that a linear relationship exists between the UTC rating and the tussive agent concentration on a log–log scale, the slope was determined with a linear regression analysis of the log–log scale. The UTC log–log slope showed no significant correlation with MMSE (r=0.10, p=0.44) (figure 5a). In a similar fashion to C_2_, C_5_ and C_u_, a significant difference in the relationship between cognitive function and C_u_ was observed in males but not in females (r=0.48, p=0.016 *versus* r=−0.16, p=0.35; as analysed by Pearson's correlation).

### Comparison of UTC among the three groups

The log–log slope of C_u_ was 0.75±0.40, 1.23±0.70 and 1.02±0.78 in the DLB, control and AD groups, respectively. It showed a tendency to be lower in the DLB group than in the control and AD groups (p=0.083, as analysed by one-way ANOVA; p_1_=0.116, as analysed by two-way ANOVA to adjust for gender) (figure 5b)

## Discussion

In this study, both cough reflex sensitivity and UTC negatively correlated with cognitive function. The cough reflex sensitivity evoking C_5_, as well as the UTC, was significantly attenuated in the DLB group. Furthermore, the DLB group showed that the UTC trend was less responsive to the increments of cough-evoking stimuli compared to the control and AD groups. The inclination of UTC between cough stimuli in males positively related to cognition, although there was no significant difference between the three groups.

Decreased cough reflex sensitivity has been established as one of the risk factors for the onset of AP in elderly people [22, 23]. Cough, as a typical respiratory symptom, can be controlled not only *via* the nucleus of the solitary tract in the brainstem but also through the cerebral cortex. Davenport [3] termed the respiratory sensation of cognitively modulated cough the UTC. The UTC plays a pivotal role in the cough motivation-to-behaviour system, leading to the emergence of respiratory sensations.

We previously demonstrated that there were both gender and age differences in the thresholds of the UTC [4, 24] and that the UTC as well as cough reflex sensitivity was attenuated in elderly patients with AP compared with healthy age-matched controls [5]. The blunted UTC is associated with the impairment of supra-medullary pathways leading to the cortex that are involved in cough reflex and the same impairment is often found in patients with cognitive deficits [25]. Unfortunately, we could not investigate the functional relationship between activated structural change in the brain and cough or the UTC in patients with dementia because it was difficult for them to keep their bodies steady in the supine position. Taking all these considerations together, we infer that the decreased UTC in DLB patients might be a hallmark that distinguishes them from patients with other dementia subtypes.

DLB and PD are both partially characterised neuropathologically by the presence of Lewy bodies and are thought to belong to the spectrum of LBD. Clinical features of DLB arise from the growth of Lewy bodies that spread from the medulla oblongata to cortical regions (including the occipital lobe) and vice versa. It was previously reported that chemosensitivity to hypoxia and hypercapnia and the sensation of dyspnoea in patients with PD were diminished [10]. Furthermore, ventilatory responses to hypercapnia were also decreased in DLB [9], suggesting that brainstem respiratory nuclei are involved in respiratory rhythmogenesis and chemosensitivity [26]. Regarding the pulmonary function of participants in this study, the decrease in FEV_1_ and FVC in patients with DLB does not contradict previous reports on pulmonary function in PD [27]. Moreover, we have previously demonstrated that the cough peak flow and the cough reflex sensitivity were decreased in patients with advanced PD [11]. Thus, the study findings are consistent with those of the aforementioned reports in terms of blunted cough reflex, deteriorated UTC and reduced sensation of the UTC. Furthermore, it has been reported that the severity of dysphagia affects the UTC ratings [28].

As cognitive function gradually deteriorates, diverse sensations to different stimuli (including pain) attenuate [29]. Our study is the first to report that cough reflex sensitivity and UTC are negatively correlated with cognitive function. It also shows that cough reflex sensitivity, particularly in females, is significantly associated with cognitive function.

Chronic hypersensitivity cough syndrome affects mainly postmenopausal women. In our previous study [30], cough reflex sensitivity due to inhalation of capsaicin in a postmenopausal animal model was significantly higher compared to control models. Another study reported that citric acid cough reflex sensitivity was significantly lower for both premenopausal and postmenopausal female patients compared to male patients [31]. Moreover, we previously reported no gender difference in the UTC threshold estimated between males and females [24]. Furthermore, evoked cough is not only a brainstem-mediated reflex response to irritation of the airways but also activates distinct areas associated with sensory, motor, cognitive, affective and motivational responses, including the posterior insula and posterior cingulate cortex [32, 33].

The MMSE is a tool that can systematically and thoroughly assess mental status. Cerebral areas such as the hippocampus, the temporal lobe, the parietal lobe and the post-dorsal part of the frontal lobe can be evaluated and as such there is partial overlap with areas responsible for cough. A previous report showed that the average cognitive scores of females declined faster with age compared to males [34].

With reference to the above, gender differences in the association between cough reflex sensitivity or UTC and cognition might be more potent in female participants than male participants. However, we found no gender role in the association between the cough reflex sensitivity of C_2_ and C_5_ in the control, AD and DLB groups.

Nociceptive stimuli are recognised as discriminative sensations by stimulating neural endings of thermal nociceptors and reaching the insular cortex through afferent pathways [35]. A recent report demonstrated that patients with DLB showed diminished volume of bilateral insulae in its early stage of development [13], whereas patients with AD showed markedly diminished volume of hippocampus in its prodromal or early developmental stage. DLB showed more deficits in the frontal, insular, somatosensory and temporal cortices, while AD showed reduced perfusion in the parietal and temporoparietal cortices [14]. Furthermore, we previously reported hypoperfusion of the insular cortex in patients with recurrent pneumonia [12]. Taking these findings into consideration, cough reflex sensitivity and UTC in patients with DLB might decrease with the advancement of hypoperfusion of the insular cortex.

In this study, the minimum required MMSE score of the cognitive function for participants was five, corresponding to the clinical level six in the functional assessment staging test [36]. We determined that patients would be responsive to stimuli that were meant to evoke UTC sensations and we would be able to distinguish the level of response to a certain extent because even patients with dementia with the clinical level of 7b can respond to pain and express comfort or willingness. One limitation of this study was that the reduced UTC may have impaired patients' ability to express severity or intractability of pneumonia.

In conclusion, both cough reflex sensitivity and UTC were attenuated in patients with DLB, compared with patients with Alzheimer's-type dementia and the age-matched healthy seniors. This result might be valuable in the treatment of patients with DLB, suggesting that early detection of pneumonia in patients with DLB is difficult and, when it is found, it is likely to have been aggravated. Moreover, further studies using chemical stimuli or those employing mechanical stimuli are needed to investigate this further.
